# Coding Psychological Constructs in Text Using Mechanical Turk: A Reliable, Accurate, and Efficient Alternative

**DOI:** 10.3389/fpsyg.2016.00741

**Published:** 2016-05-30

**Authors:** Jennifer Tosti-Kharas, Caryn Conley

**Affiliations:** ^1^Management, Babson CollegeBabson Park, MA, USA; ^2^Information, Risk, and Operations Management, McCombs School of Business, University of Texas at AustinAustin, TX, USA

**Keywords:** content analysis, coding, qualitative research methods, Mechanical Turk, crowdsourcing

## Abstract

In this paper we evaluate how to effectively use the crowdsourcing service, Amazon's Mechanical Turk (MTurk), to content analyze textual data for use in psychological research. MTurk is a marketplace for discrete tasks completed by workers, typically for small amounts of money. MTurk has been used to aid psychological research in general, and content analysis in particular. In the current study, MTurk workers content analyzed personally-written textual data using coding categories previously developed and validated in psychological research. These codes were evaluated for reliability, accuracy, completion time, and cost. Results indicate that MTurk workers categorized textual data with comparable reliability and accuracy to both previously published studies and expert raters. Further, the coding tasks were performed quickly and cheaply. These data suggest that crowdsourced content analysis can help advance psychological research.

## Introduction

Crowdsourcing is a growing phenomenon in which distributed workers perform microtasks for compensation online (Howe, 2006). Amazon's Mechanical Turk (MTurk) website is a popular example, reporting over 500,000 registered workers (commonly called “Turkers”) in more than 190 countries with over 300,000 available tasks (called Human Intelligence Tasks, or HITs) at any given time (MTurk Census, 2011). Given the size and diversity of the registered Turkers, MTurk has emerged as a useful tool for a variety of academic research purposes, such as data categorization (e.g., Kittur et al., 2008, 2009; Alonso and Mizzaro, 2009; Wang et al., 2015), recruiting psychological experiment subjects (Buhrmester et al., 2011; Goodman et al., 2012; Mason and Suri, 2012; Paolacci and Chandler, 2014), and replicating previous research results (Horton et al., 2010; Paolacci et al., 2010; Buhrmester et al., 2011; Wang et al., 2015).

One area in which crowdsourced workers have the potential to facilitate psychological research, but which remains largely untapped, is in content analytic coding. Content analysis is an important methodology in psychological research, allowing qualitative data to be coded for underlying constructs of interest (e.g., Weber, 1990; Krippendorff, 2004). For example, content coding is commonly used in the area of personally-written life stories, in which identity construction is seen as a narrative task (e.g., McAdams et al., 2001). Researchers have analyzed the content of self-narratives along numerous dimensions—such as plot sequence, coherence, closure, and emotional tone—which in turn relate to constructs such as psychological well-being and ego development (e.g., King et al., 2000; McAdams et al., 2001; Pals, 2006; Adler et al., 2007; Adler and Poulin, 2009). These studies typically employ multiple coders who are screened, hired, and trained. Alternatively, researchers may use computer software programs, such as Linguistic Inquiry and Word Count (LIWC) (e.g., Pennebaker et al., 2007; Hirsh and Peterson, 2009), to code narratives. However, the process of coding by hand is both time- and labor-intensive, while computerized coding lacks the interpretive ability of human coders (Weber, 1990; Krippendorff, 2004). By improving the efficiency and cost effectiveness of performing content analysis without sacrificing reliability and accuracy, the quantity, sources, and types of data for analysis would expand. In turn, this analytical flexibility could allow researchers to explore novel and nuanced research questions and further the field of psychology. In this paper, we evaluate crowdsourcing for content analysis of well-established psychological constructs in terms of efficiency, cost effectiveness, reliability, and accuracy.

Crowdsourced coders combine the benefits of coding by hand and computer-aided coding. Crowdsourced workers are readily available and can be recruited, trained, and compensated for a low cost, approaching the ease and flexibility of computer software. Unlike computerized solutions, these workers have sophisticated interpretive ability to decipher textual elements like context, ambiguous pronouns, and figures of speech. Despite their potential to aid in content coding, researchers have underutilized Turkers in this capacity. One recent study found that Turkers were able to reliably and accurately classify social media posts on Twitter, known as “tweets,” according to nine topic categories pertaining to diabetes (Harris et al., 2015). In this study, the reliability of Turkers' ratings ranged between 0.62 and 0.84 across 10 coding dimensions, meaning all were in good to excellent reliability ranges. In other research Turkers proved as capable as trained researchers in applying inductively generated codes to text (Conley and Tosti-Kharas, 2014). Here, Turkers' interrater reliabilities ranged from 0.56 to 0.80 across 6 coding dimensions, with only 1 dimension falling outside the good to excellent range of 0.60 or higher. Yet these studies both operated outside the field of psychology.

To assess the capability of MTurk to aid content analysis in psychological research, we followed several steps. First, we presented Turkers with published, validated psychological coding schemes which we asked them to apply to personally-written stories collected by the authors. We next evaluated the reliability and accuracy of Turkers' codes compared to published studies, expert ratings, and computer software. Further, we assessed the cost of coding via MTurk in terms of time and money. We conclude by discussing the implications of using MTurk for coding self-narratives in psychological research and outlining several best practices for using this approach.

## Methods

### Participants and procedures

We registered on MTurk as “requesters,” meaning we could post-tasks (HITs) for completion. We presented Turkers with what can best be described as brief personally-written self-narratives, or the life stories people tell that reflect their unique identities and personalities (e.g., McAdams et al., 2001; Hirsh and Peterson, 2009). The authors collected these stories in previous unpublished research using a Web-based survey of working adults. Participants described an event from the previous 12 months that greatly influenced their career perceptions. The 30 passages used in this study focused on a specific incident and its implications (e.g., King et al., 2000), and ranged in length from one sentence to three paragraphs. An example of a passage used in this study follows:

“Last year I left my job and relocated with my family. I have been looking for a new job over the past year and am still looking. The experience has made me reflect more on what I would like to do. I am looking for a similar position to my last job but am at the same time considering other alternatives.”

Turkers read a passage in its entirety and then coded the passage by assigning categories based on one of the theoretical dimensions of interest. The coding unit was the entire passage. We chose four dimensions commonly used and previously validated in psychological research on self-narratives (e.g., King et al., 2000; McAdams et al., 2001; Pals, 2006; Adler and Poulin, 2009). These dimensions were: redemption and contamination, closure, overall emotional tone, and discrete emotions. Turkers assigned the coding category that they believed best applied to that specific passage. We present a detailed description and coding scheme for each of the dimensions below.

The number of passages Turkers coded (either 1 or 5 narratives) and the number of available coding categories (ranging from 1 for closure to 10 for discrete emotions) varied per HIT. The HIT pay rate increased with the number of passages and coding categories, ranging from $0.02 to $0.12 per HIT, a payment rate that is in line with previous studies (e.g., Conley and Tosti-Kharas, 2014; Harris et al., 2015). To encourage honest work, we indicated that HITs would be accepted, meaning payment made, only if their responses approximately matched those of the other Turkers rating the same passage. Turkers coded each passage only once; however, they could code different passages by completing additional HITs. We aimed to have each passage coded by 10 unique coders on each of the four theoretical dimensions. All Turkers were eligible to complete the HITs, provided that they had a 95% HIT acceptance rate based on prior work performed on MTurk and were based in the United States. Overall, 124 unique Turkers completed 404 HITs in this study, for approximately 10 ratings per passage. We present a sample HIT description and instructions in Appendix A. Task descriptions and instructions for the complete set of HITs used in this study are available from the authors upon request.

### Coding dimensions

#### Redemption and contamination

Redemption and contamination refer to the plot sequences of self-narratives involving a transformation or change (McAdams et al., 2001). In redemption sequences, life events progress from a bad, emotionally negative state to a good, emotionally positive one, while in contamination sequences events progress from good to bad or from bad to worse (McAdams et al., 2001). We asked Turkers to indicate whether each self-narrative contained a redemption sequence (yes/no) and a contamination sequence (yes/no). McAdams et al. (2001) developed and validated these categories; however, we revised some of the wording to be more colloquial, given that Turkers were not trained researchers. Prior to completing the HIT, we provided Turkers with example passages in the task instructions to illustrate when redemption and contamination sequences should be coded.

#### Closure

Closure refers to the degree of resolution expressed by narrators, where complete resolution implies that there are no outstanding issues or emotions remaining to be resolved. Turkers rated the degree of closure expressed in each self-narrative via a single item used in previous research (e.g., King et al., 2000; Pals, 2006; Adler and Poulin, 2009) from 1 (very unresolved) to 5 (completely resolved). We provided Turkers with the definition of closure, but did not provide example passages to illustrate the use of codes.

#### Emotional tone

The overall emotional tone of self-narratives reflects the general positive or negative nature of the narrator's writing (e.g., McAdams et al., 1997). Turkers rated the overall emotional tone of each passage using a single-item measure used in previous research (McAdams et al., 1997; Adler and Poulin, 2009) from 1 (completely negative and pessimistic) to 5 (completely positive and optimistic). We did not provide Turkers with example passages to illustrate these codes.

#### Discrete emotions

Self-narratives can also be evaluated in terms of the narrator's display of specific emotions, such as excitement or distress. Although this is not a common approach in self-narrative coding, we wanted to see whether Turkers could evaluate the distinct emotions expressed in self-narratives using the Positive and Negative Affect Schedule (PANAS, Watson and Clark, 1984; Watson et al., 1988). PANAS is commonly used to assess the display of discrete emotions and has been found to be stable for 2 months. Although it has not been typically used in coding retrospective events that may have occurred up to 12 months prior, we chose to use it because it is an established scale that we thought would provide valuable nuance in understanding the emotions expressed. We measured positive affect (PA) and negative affect (NA) using 10 items from a 20-item measure designed to load on separate, orthogonal dimensions (Watson et al., 1988). We chose the 10 items based on those we thought would be most relevant given the content of the passages to be coded. The 5 PA items included excited, strong, enthusiastic, proud, and inspired, and the 5 NA items were distressed, upset, guilty, scared, and ashamed. Turkers rated the extent to which the narrator expressed each emotion on a 5-point Likert-type scale, from 1 (very slightly or not at all) to 5 (extremely). Again, we did not provide Turkers with example passages to illustrate these codes.

## Results

### Task completion and acceptance

The time required to complete all HITs was 17.5 days. The total cost was $19.00. Table 1 provides detailed information about the different HITs we posted to MTurk, such as number of HITs requested and number of passages per HIT; percentage of HITs completed by Turkers; percentage of HITs accepted, meaning workers were paid for successful completion; number of unique Turkers completing HITs; and average time to complete a HIT. Our HIT acceptance policies varied depending on the type of task, as summarized in Appendix B.

**Table 1:** **Summary task information**.

	**Redemption^a^**		**Emotional Tone^a^**			**Closure**	**Discrete emotions**
	**Round 1**	**Round 2**	**Round 1**	**Round 2**	**Round 3**		
**HITs**							
HITs requested	200	40	200	40	40	40	100
HITs completed	117 (59%)	40 (100%)	55 (28%)	12 (30%)	40 (100%)	40 (100%)	100 (100%)
HITs accepted	93 (79%)	32 (80%)	47 (85%)	11 (92%)	37 (93%)	36 (90%)	83 (83%)
Passages per HIT	1	5	1	5	5	5	1
HITs analyzed	102 (87%)	35 (88%)	41 (75%)	9 (75%)	38 (95%)	39 (98%)	90 (90%)
**TURKERS**							
Unique Turkers	24	27	10	7	27	27	22
HITs per Turker	4.88	1.48	5.50	1.71	1.48	1.48	4.55
Turkers with rejected HITs	13	6	3	1	3	3	5
**TIME**							
Average seconds to complete (median)	69.96 (48)	233.5 (190.5)	50.87 (33)	164.17 (120.5)	147.85 (109)	157.08 (128.5)	114.65 (85.5)
Total time task was available (hours: minutes: seconds)	96:43:48	53:37:03	54:47:01	23:08:51	71:28:25	96:52:36	23:48:29
**COMPENSATION**							
Amount paid per HIT	$0.02	$0.12	$0.01	$0.06	$0.10	$0.12	$0.05
Effective hourly rate	$1.03	$1.85	$0.71	$1.32	$2.43	$2.75	$1.57
Total paid to Turkers	$1.86	$3.84	$0.47	$0.66	$3.70	$4.32	$4.15

a *Redemption and Emotional Tone have multiple rounds of coding in order to achieve 100% HIT completion rate. We adjusted the effective hourly rate as well as number of passages coded per HIT to encourage Turkers to complete these tasks*.

### Exclusion criteria

Before analyzing the data collected, we identified poor-quality data to exclude from our analyses for this study. This process differed from the process used to determine whether to pay Turkers for their work (per HIT acceptance policy above). We followed two rules to exclude poor-quality responses from our analyses. First, we excluded responses where Turkers clearly did not follow HIT directions, for example, they only provided ratings for 3 of the 10 emotions. Second, we excluded responses for which completion times were extremely short. For example, we excluded responses where Turkers spent 10 s or less to read the passage and provide rating(s) (i.e., 10 s for a single passage, 50 s for five passages). We believed this was not enough time to read and then accurately code the data. We did not want to bias our reliability and accuracy results by including only codes that agreed with the majority, nor did we wish to exclude honest coding attempts. Accordingly, we included responses in our analyses and results regardless of whether they had met our HIT acceptance policies, provided that they met these two criteria for high-quality responses. Table 1 presents the total responses analyzed in each task.

### Reliability and accuracy

To assess the inter-rater reliability of Turkers' ratings we calculated the Intraclass Correlation Coefficient (ICC), the recommended procedure for assessing inter-rater reliability for multiple raters (Shrout and Fleiss, 1979). Overall, the ICC2 values among Turkers were high, ranging from 0.72 (PA) to 0.95 (emotional tone). In fact, Turkers' reliabilities were comparable to those reported in prior studies using the same measures, ranging from 0.72 (closure) to 0.87 (PA). Table 2 presents a summary of reliability results.

**Table 2:** **Intraclass correlation coefficients (ICC)^a^**.

	**Turker Ratings^b^**	**Expert Ratings**	**Accuracy of turker ratings**	**Published research^c^**
Redemption		0.89 (20)	0.95 (20)	0.79
Round 1	0.79 (102)	0.78 (10)	1.00 (10)	
Round 2	0.89 (180)	1.00 (10)	0.89 (10)	
Contamination		0.82 (20)	0.89 (20)	0.78
Round 1	0.79 (102)	0.72 (10)	0.89 (10)	
Round 2	0.93 (180)	0.90 (10)	0.90 (10)	
Closure	0.89 (194)	0.77 (10)	0.76 (10)	0.72
Emotional tone		0.90 (30)	0.88 (30)	0.85
Round 1	0.81 (41)	0.79 (10)	0.80 (10)	
Round 2	0.92 (45)	0.93 (10)	0.95 (10)	
Round 3	0.95 (190)	0.94 (10)	0.88 (10)	
Positive affect	0.72 (448)	0.72 (100)	0.77 (100)	0.87
Enthusiastic	0.76 (90)	0.66 (20)	0.86 (20)	
Excited	0.75 (90)	0.92 (20)	0.80 (20)	
Inspired	0.67 (89)	0.73 (20)	0.75 (20)	
Proud	0.62 (90)	0.63 (20)	0.72 (20)	
Strong	0.48 (89)	0.62 (20)	0.53 (20)	
Negative affect	0.78 (450)	0.88 (100)	0.81 (100)	0.80
Ashamed	0.74 (90)	0.94 (20)	0.70 (20)	
Distressed	0.79 (90)	0.83 (20)	0.84 (20)	
Guilty	0.50 (90)	0.65 (20)	0.74 (20)	
Scared	0.27 (90)	0.09 (20)	0.61 (20)	
Upset	0.84 (90)	0.95 (20)	0.96 (20)	

a *We report ICC2 for Turker ratings and ICC1 statistics for expert ratings and accuracy between Turkers and experts*.

b *Values in parentheses indicate number of HITs*.

c *The studies included in this analysis were: Adler and Poulin (2009), King et al. (2000), McAdams et al. (1997), McAdams et al. (2001), Pals (2006). We averaged the ICCs from these studies*.

As an additional indicator of the quality of Turkers' codes, we evaluated whether ratings of the 10 discrete emotions maintained their intended dimensionality. A principal components factor analysis using Varimax rotation showed that two factors accounted for 71.83% of the variance. The two factors corresponded to the PA and NA dimensions with high scale Cronbach's alpha coefficients (PA: 0.93, NA: 0.84). These reliability scores are comparable to those reported in previous research (PA: 0.88, NA: 0.87) (Watson et al., 1988).

To assess the accuracy of the Turkers' work, we compared their ratings to those of trained experts as well as computer software. The two authors, both academics who have published research in this area, provided expert ratings by coding the same narratives on the same dimensions. The experts' overall ICC1 values for the different tasks were high, ranging from 0.72 (PA) to 0.90 (emotional tone), and comparable to reliability among Turkers. We then compared Turkers' ratings to the expert ratings for accuracy. For redemption and contamination, we used the majority of Turkers' ratings as the basis for comparison, while for emotional tone, closure and discrete emotions, we compared the mean Turkers' ratings to the expert ratings. ICC1 values across tasks were very high, ranging from 0.76 (closure) to 0.95 (redemption). Overall, these results suggest Turkers provided ratings consistent with the ratings of both other Turkers and experts.

Since text analysis software has been used in self-narrative research (e.g., Hirsh and Peterson, 2009), we compared the ratings of emotions provided by Turkers to the output of the LIWC software program (Pennebaker et al., 2007; http://www.liwc.net/). We examined emotions because this was the only coding dimension in our study with a predefined LIWC dictionary. We calculated the overall “emotional tone” generated by LIWC as the difference between the percentage of positive emotion words and the percentage of negative emotion words. For discrete emotions, we calculated a Turker average positive rating and a Turker average negative rating per passage, which we compared to the LIWC percentage of words denoting positive and negative emotions. We compared correlations between the LIWC output, the average Turkers' ratings, and the average experts' ratings across self-narrative passages.

The correlations between LIWC output and both average Turkers' ratings and expert ratings were positive, but not statistically significant (*r*s ranged between 0.15 and 0.40, *ns*). By contrast, the correlations between the Turkers' ratings and expert ratings were positive and statistically significant (*r*s ranged between 0.77 and 0.85, *p* < 0.001). These results suggest that substituting LIWC for human raters to identify both the overall emotional tone and positive and negative affect of self-narrative passages in this study is not an ideal solution. Appendix C presents an illustration of the differences between LIWC output and human interpretation.

## Discussion

We examined the suitability of MTurk for performing content analysis of personally-written text data using established psychological codes. Turkers coded self-narratives for redemption and contamination sequences, degree of closure, and affective tone, both in general and for specific emotions. The findings indicate that these non-expert workers coded the data with reliability and accuracy comparable to both published studies and trained experts. It appeared Turkers outperformed existing computerized software. Further, the coding was completed efficiently, typically within a few days, and at low cost, in this case $19.00 total. Thus, coders recruited via crowdsourcing in general, and MTurk in particular, appear to combine the efficiency of computer software with the sophisticated understanding of human coding.

Using MTurk for content analysis of personally-written text collected via interview, survey, or archives may advance psychological research in several ways. MTurk is low-cost compared to traditional means of recruiting and training coders in terms of time and money. Thus, MTurk provides greater theoretical flexibility compared to traditional content analytic approaches. The abundant supply of Turkers enables researchers to quickly analyze large datasets and apply and refine numerous coding schemes simultaneously. Turkers are relatively diverse and geographically distributed (e.g., Ross et al., 2010; Iperiotis, 2015; also see http://demographics.mturk-tracker.com); therefore, their codes are likely to be truly independent from each other and from researchers. Unlike research assistants, we do not reasonably expect Turkers to have a priori knowledge of the psychological theories or researchers involved, so their responses are unlikely to be biased. Since Turkers code only a small subset of the entire dataset (e.g., 1 or 5 passages in our study), they are unlikely to experience fatigue. Researchers can collect multiple rounds of coding and assess the concordance between those rounds. Further, unlike computer software, Turkers are humans capable of sophisticated interpretation of text, including context, idioms, and other ambiguities. Researchers have recently been taking advantage of the proliferation of the Internet and social media, including publically available Facebook and Twitter posts, personal blogs, and customer reviews. MTurk can help scholars leverage the increasingly available data by providing access to cheap yet high-quality thematic coding.

Of course, MTurk is not without its disadvantages. Where possible, we offer our suggestions for how to deal with these issues, if not eliminate them entirely. First, researchers cannot follow up with Turkers to clarify their codes. Accordingly, researchers could require Turkers to provide explanations for chosen coding categories. Second, Turkers may try to game the system to provide careless, dishonest, or bogus responses (e.g., Rzeszotarski and Kittur, 2011). We recommend designing HIT acceptance policies to solicit genuine effort (e.g., Kittur et al., 2008). For example, we did not pay Turkers whose ratings differed dramatically from other ratings. Instructional manipulation checks may also be helpful in determining whether Turkers are paying close attention to coding instructions (Oppenheimer et al., 2009). While we provided minimal training, researchers can design extensive training, or require pre-qualification tasks, such as applying codes accurately and consistently, before allowing Turkers to complete HITs. In addition, researchers should design HITs to facilitate Turkers' comprehension and pre-test coding categories and wording of instructions. Future studies should consider whether MTurk is effective for other types of coding schemes and other forms of data. Finally, studies of Turker demographics show that although there is diversity a    Round age, gender, marital status, educational level, and income (Ross et al., 2010; Iperiotis, 2015), Turkers are generally younger and with higher education levels than the general population. That is, they may be more reflective of the U.S. Internet-using population than the U.S. population as a whole (Ross et al., 2010). Researchers should take into account the potential for bias as a result of these demographics. It would be helpful to assess some basic demographics about the Turkers performing coding tasks to gauge the extent to which bias might be present. Earlier, we mentioned that Turkers likely do not have psychological training or experience. This, too, should be assessed so that any bias can be considered.

In addition, the low pay rates associated with MTurk raise issues of ethical treatment of Turkers. This issue has been discussed at length in other studies recruiting Turkers as research participants (e.g., Horton et al., 2010; Paolacci et al., 2010; Buhrmester et al., 2011). We echo calls for researchers using MTurk to be mindful of fair and ethical standards when designing HITs and compensating workers. It may be better to err on the side of offering payment for a sincere effort, even if the response differs from what the researcher expected. In addition, we experimented with how much Turkers were paid across multiple rounds of requesting HITs. We found that Turkers would not complete HITs if they did not feel the pay was sufficient.

We caution that although MTurk provides fast, inexpensive coding, it is certainly not appropriate for all content or narrative analytic approaches. Psychologists performing qualitative analysis must first and foremost spend time understanding their data, perhaps especially when involving self-narrative data. Therefore, MTurk coding does not substitute a researcher's deep understanding. Further, MTurk is ideal for shorter text passages, like the short self-narratives used in this study, or the tweets employed in other studies. While a longer passage could be divided into smaller segments, valuable context may be lost.

Another significant issue with using primary data is ensuring the privacy of the original participants who provided the text for coding. This issue becomes especially relevant when the text passages are self-implicating, as in the case of self-narratives. Often researchers ask people to write about difficult, even traumatic instances in their lives, which they may be reticent to share with others (e.g., King et al., 2000; Pals, 2006). Researchers should therefore follow similar protocol to preparing these data to be coded by student assistants or published, such as removing any personally identifying detail. In fact, we strongly suggest that researchers remove identifying detail to the extent that even someone who knows the person's situation would not be able to identify the author of the passage. In situations where this is not possible, researchers should refrain for using MTurk for content coding. In reality, the likelihood of a Turker recognizing the author of a passage is much less than when both authors and coders are recruited from the same university or geographic area. To go a step further, participants' stories that were solicited by the researchers would not be posted for coding on MTurk or any other crowdsourcing platform without participants' express consent. It is up to the individual researcher's Institutional Review Board concerning the ethical treatment of human subjects to determine the precise protocol. However, for the current study, the researchers explained on the consent document that a distributed team of coders may also have access to the data. This concern is mitigated when researchers are using archival data that has already been made public, as in newspapers, magazines, and other Internet sources. At the same time, data posted on MTurk HITs is not truly “public” in the same way that information posted on publicly accessible Internet sites is. Rather, Turkers are first screened by MTurk to qualify to perform HITs, and then they must log in to view HITs. It is not clear that Turkers would have the incentive or rationale to attempt to save and reuse or republish the passages they are coding. Even if they wished to do so, there is not an easy way to save or transfer data from within a HIT. In addition, Turkers would never see a complete dataset, so there are limits to the breadth of data they would see. In sum, although the ethical concern in having non-trained crowdsourced workers assist in content coding of personally-written stories is non-trivial, we believe it can be overcome with careful attention by the researchers.

## Conclusion

Content analysis provides valuable insights into human psychology, whether personality, cognition, and/or emotion. However, content coding can be a lengthy, costly, arduous process for researchers. If traditional content analysis approaches can be improved upon, particularly regarding efficiency and cost, researchers will be able to expand the possible research questions that can be pursued and breadth of data that can be analyzed, which can further their field of knowledge. This paper aims to advocate for and support content coding in psychological research by presenting crowdsourcing using Amazon's Mechanical Turk as a way to perform content coding that is more time efficient and cost effective than traditional methods, with no demonstrable deficit in reliability and accuracy.

## Author contributions

JT contributed to this manuscript in the areas of research design, data collection, data analysis, manuscript writing, and editing. She performed more of the manuscript preparation than did CC. CC contributed to this manuscript in the areas of research design, data collection, data analysis, manuscript writing, and editing. The project was initially her idea.

### Conflict of interest statement

The authors declare that the research was conducted in the absence of any commercial or financial relationships that could be construed as a potential conflict of interest.

## Appendix A

### Sample task and instructions: emotional tone

**Title:** Categorize the emotional tone of stories about significant events in people's careers.

**Description:** We're trying to understand how professionals in today's economy respond to significant events in their careers and we need your help! In this task, you rate the event based on its emotional tone.

**Instructions:** Read a short story where someone describes an event that happened in his or her work life in the past year, then rate the story's overall emotional tone on a scale from *completely negative and pessimistic* (1) to *completely positive and optimistic* (5).

**How HITs are accepted:** In order to accept your HIT, we will compare your ratings for the story with the average of other Turkers who have rated the same story. **If your rating is “close” to the average rating, your HIT will be accepted**. The emotional tone will be assigned a number of points: *completely negative and pessimistic* (1), *somewhat negative and pessimistic* (2), *neither negative nor positive* (3), *somewhat positive and optimistic* (4), *completely positive and optimistic* (5). Three examples illustrate how the comparison for acceptance is made:

1. If the average response for your story is 3.5, then your rating is considered “close” to the average if you select either *neither negative nor positive* (3) or *somewhat positive and optimistic* (4).
2. If the average response for your story is 1.2, then your rating is considered “close” to the average if you select either *completely negative and pessimistic* (1) or *somewhat negative and pessimistic* (2).
3. If the average response for your story is 4.7, then your rating is be considered “close” to the average if you select either *somewhat positive and optimistic* (4) or *completely positive and optimistic* (5).

Therefore, in the above examples, if you were “close” to the average response for your story, then you would receive payment the HIT. We appreciate your hard work, and will reward you with a 10% bonus if your HIT acceptance rate with us is 90% or higher.

### Your task

#### Instructions:

Please read the following story and rate how negative or positive the overall emotional tone of the story is using the scale below.

#### Story:

On a recent transcontinental flight I heard the flight attendant respond to a passenger complaining about the smell coming from the bathroom area. The flight attendant said that it always smells bad. As a former aviation executive it sickened me to see how much my beloved industry had declined. It made me realize that I can never go back.

**Table:**

How would you rate the overall emotional tone of this story? (required)	*Completely negative and pessimistic (1)*	*Somewhat negative and pessimistic (2)*	*Neither negative nor positive (3)*	*Somewhat positive and optimistic (4)*	*Completely positive and optimistic (5)*

Please provide any additional comments you may have below. We appreciate your input!

## Appendix B

### Hit acceptance policies

**Table:**

	**Narratives per HIT**	**Acceptance criteria**
Redemption and contamination		
Round 1	1	At least 1 rating matched the Turker majority rating^a^
Round 2	5	At least 6 of 10 ratings matched the Turker majority
Closure	5	At least 4 of 5 ratings were within ±1 *SD* of the mean Turker rating
Emotional Tone		
Round 1	1	Ratings were within ±1 *SD* from the mean Turker rating, rounded up or down^b^
Rounds 2 and 3	5	At least 4 of 5 ratings were within ±1 *SD* of the mean Turker rating
Discrete Emotions	1	At least 8 of 10 ratings were within ±1 *SD* of the mean Turker rating

a *Since we used majority and mean Turker ratings as part of our HIT acceptance criteria, we could only accept HITs and therefore compensate Turkers once we had collected all ratings. Turkers generally prefer to be paid promptly. While our approach led to a slight delay in the time to HIT acceptance, it was still within reasonable and acceptable limits of payment*.

b *For example, if M = 2.53 and SD = 1.45, the range for acceptance was 1.08–3.98, so we accepted ratings of 1–4*.

## Appendix C

### Comparison of LIWC output and human coders

To illustrate the differences between the LIWC output and human coders, we offer two example self-narratives. People, either recruited through MTurk or the experts in this study, applied codes ranging from 1 (completely negative and pessimistic) to 5 (completely positive and optimistic). The percentage of words identified by LIWC as evoking positive emotion ranged between 0 and 6.9% while the percentage of words identified as evoking negative emotion ranged between 0 and 5.08% per self-narrative.

*Example 1*: As a director for my company I am charged with providing benefits that are cost effective for the company while providing financial protection for employees. The company continued slashing, reducing, and cutting benefits (that's redundant I know!). It got to the point where though I was responsible for the company's benefits program, I was embarrassed by the benefits program! It wasn't something I could honestly support and communicate to our employees. When the company decided to relocate, I had no qualms about not moving with them.

In the first example, LIWC identified this self-narrative as the most positive of all self-narratives analyzed (6.9% positive words) and not at all negative (0% negative words). Based on this output, we would expect human coders to rate the emotional tone toward the higher, more positive end of the response scale. However, the human coders rated the self-narrative as extremely negative (Turkers: 1.66 and Experts: 1), the opposite of the LIWC output. Examination of the phrasing helps to explain the ratings discrepancy. Several phrases convey the overtly negative tone, such as, “I was embarrassed by the benefits program,” “it wasn't something I could honestly support,” and “I had no qualms about not moving with them.” These phases lack words evocative of negative emotion, but the context allows the reader to infer a negative sentiment. The word counts used by LIWC do not consider the broader context or sophisticated grammatical patterns, such as double-negatives. A second example further illustrates the discrepancy between the ratings provided by LIWC and human coding.

*Example 2*: I was recently offered a voluntary early retirement package with an enhanced pension, significant medical insurance subsidy, and severance payments. I looked at the numbers and realized that retirement was feasible. I took the package and retired 2 months ago. From day one I have not missed working. I realize now that my identity is not tied up in my job anywhere near as much as I thought it would be. My identity now is as someone who could retire at 60 years old!

The LIWC software identified this self-narrative as not at all positive (0% positive words) and slightly negative (1.19% negative words). We would therefore expect the human coders to report codes tending toward the low end of the response scale. However, the human coders evaluated the self-narrative to be extremely positive (Turkers: 4.5 and Experts: 5), again the opposite of the LIWC results. A human reader would likely pick up on the excitement and optimism indicated by phrases such as, “From day one I have not missed working,” and “My identity now is as someone who could retire at 60 years old!” However, this interpretation requires consideration of the relevant context of modern working life—for example, knowing the typical retirement age—and would be difficult to replicate based on word count alone. In sum, these two examples help to illustrate the shortcomings of one popular text analysis tool, LIWC, relative to humans in coding emotional tone in self-narratives.
